# Type 1 diabetes alters lipid handling and metabolism in human fibroblasts and peripheral blood mononuclear cells

**DOI:** 10.1371/journal.pone.0188474

**Published:** 2017-12-04

**Authors:** Albert R. Jones IV, Emily L. Coleman, Nicholas R. Husni, Jude T. Deeney, Forum Raval, Devin Steenkamp, Hans Dooms, Barbara S. Nikolajczyk, Barbara E. Corkey

**Affiliations:** 1 Obesity Research Center, Evans Department of Medicine, Boston University School of Medicine, Boston, MA, United States of America; 2 Yale University School of Medicine, New Haven, CT, United States of America; 3 Department of Microbiology, Boston University School of Medicine, Boston, MA, United States of America; 4 Endocrinology Section, Evans Department of Medicine, Boston University School of Medicine, Boston, MA, United States of America; 5 Rheumatology Section, Evans Department of Medicine, Boston University School of Medicine, Boston, MA, United States of America; 6 Department of Translational Research in Diabetes, University of Kentucky School of Medicine, Lexington, KY, United States of America; 7 Department of Pharmacology and Nutritional Sciences, University of Kentucky School of Medicine, Lexington, KY, United States of America; Baylor College of Medicine, UNITED STATES

## Abstract

Triggers of the autoimmune response that leads to type 1 diabetes (T1D) remain poorly understood. A possibility is that parallel changes in both T cells and target cells provoke autoimmune attack. We previously documented greater Ca^2+^ transients in fibroblasts from T1D subjects than non-T1D after exposure to fatty acids (FA) and tumor necrosis factor α (TNFα). These data indicate that metabolic and signal transduction defects present in T1D can be elicited *ex vivo* in isolated cells. Changes that precede T1D, including inflammation, may activate atypical responses in people that are genetically predisposed to T1D. To identify such cellular differences in T1D, we quantified a panel of metabolic responses in fibroblasts and peripheral blood cells (PBMCs) from age-matched T1D and non-T1D subjects, as models for non-immune and immune cells, respectively. Fibroblasts from T1D subjects accumulated more lipid, had higher LC-CoA levels and converted more FA to CO_2_, with less mitochondrial proton leak in response to oleate alone or with TNFα, using the latter as a model of inflammation. T1D-PBMCs contained and also accumulated more lipid following FA exposure. In addition, they formed more peroxidized lipid than controls following FA exposure. We conclude that both immune and non-immune cells in T1D subjects differ from controls in terms of responses to FA and TNFα. Our results suggest a differential sensitivity to inflammatory insults and FA that may precede and contribute to T1D by priming both immune cells and their targets for autoimmune reactions.

## Introduction

Type 1 Diabetes (T1D) is an autoimmune disease with a genetic predisposition that primes the immune system, mainly T cells, to destroy the insulin-producing β cells of the pancreas. Despite the strong evidence for a genetic predisposition towards T1D, epidemiological data indicating the involvement of additional environmental factor(s) in disease etiology remain compelling. The human leukocyte antigen (HLA) genotype, the dominant genetic marker for T1D, is found in only 20–30% of T1D patients, and in only 50% of patients diagnosed in early childhood [1, 2]. In addition, less than half of HLA-susceptible monozygotic twins both develop T1D [3, 4].

Studies that focus on distinct environmental triggers of T1D support a combination of inflammatory and metabolic changes that associate with disease onset/progression. For example, identical twins discordant for T1D show a significant difference in the expression of genes of arachidonic acid metabolism and TGF-β signaling, two key pathways involved in inflammation [3, 4]. Specific inflammatory cascades may be critical for T1D onset, as evidenced by data suggesting that viral infection and the associated inflammation can precede T1D [5, 6], and exposure of human pancreatic islets to coxsackie virus B5 or inflammatory cytokines increases the expression of innate immune receptors [7, 8] that may trigger a feed-forward inflammatory loop. Furthermore, viral infection induces chronic pancreatic inflammation that preferentially produces CD8-mediated immune responses implicated in β cell destruction [7, 8].

Both environmental cues and genetic predispositions impact expression of cytokines implicated in final stages towards T1D including tumor necrosis factor α (TNFα) [9], a cytokine activated by multiple viruses and broadly implicated in anti-viral immune responses [10]. Numerous inflammatory cytokines, including TNFα, are produced at higher concentrations in diabetic subjects compared to controls [11–13]. TNFα is required for β-cell destruction, likely due to its ability to induce endoplasmic reticulum stress and to promote the accumulation and activation of immune cells in pancreatic β-cells [14–16]. Finally, TNFα deletion in NOD mice, a strain widely used as a model for T1D, protects against β cell destruction and associates with the absence of insulin-targeted T-cells [17].

One mechanism by which inflammation may impact cellular metabolism in T1D is through complex relationships between TNFα and multiple metabolic processes. TNFα inhibits FA oxidation and stimulates lipolysis in adipocytes [18]. In contrast, TNFα stimulates lipid synthesis and secretion in hepatocytes [19–22]. FA, in turn, have been shown to induce reactive oxygen species (ROS) production in many cells including PBMCs [23] and endothelial cells, leading to increased activation of TNFα genes, which have been linked to the production and release of ROS in fibroblasts in fuel a feed-forward inflammatory loop [24].

Cells share similar pathways of lipid metabolism and cytokine signaling, and we anticipated that abnormalities in these pathways, if present in T1D patients, would be broadly apparent. To address the links among inflammation, cellular metabolism and alterations in cellular responses in T1D, we quantified the metabolic differences between primary human T1D and non-T1D control cells, using dermal fibroblasts as a model of non-immune cells and PBMCs as a model of immune cells. We demonstrated that T1D cells differ in cellular metabolism while resting and while responding to FA (oleate) and cytokines (TNFα) compared to cells from non-T1D subjects. These findings raise the possibility that T1D etiology is contingent on metabolic differences that trigger the pathogenic autoimmune response and/or the “attack me” signal responsible for β cell destruction in genetically susceptible individuals.

## Research design and methods

### Ethics statement

De-identified human fibroblasts were obtained from The Coriell Institute for Medical Research (Camden, NJ), under an exempt Institutional Review Board (IRB) protocol. Dermal fibroblasts were obtained from 5 five normal subjects and five subjects diagnosed with T1D, and technical replicates of cells were assayed. All 10 donors were white males between the ages of 17 and 20 years old. Following informed consent under a Boston University IRB-approved protocol, human peripheral blood (50–100 mL) was collected from T1D or non-T1D subjects by venous puncture into acid/citrate/dextrose-containing tubes and cells were processed into PBMCs as described (Jagannathan-Bogdan et al, 2011 J of Immunol). T1D subjects were recruited from the Endocrinology, Diabetes and Nutrition Clinic at BMC. Additional T1D and non-T1D donors were recruited from the Clinical Research Center and the Boston University School of Medicine community. Both human tissues were handled solely by the authors of this paper in our country of residence.

### Cell cultures

Fibroblasts were grown in Minimal Essential Medium (MEM) supplemented with 2X concentration of essential and non-essential amino acids for MEM, 1X MEM vitamin solution, 1X antibiotic/antimycotic, and 10% fetal bovine serum from Hyclone Laboratories, Inc. All other cell culture solutions were purchased from Invitrogen. Upon confluence, cells were removed from culture flasks by incubating for 1 minute in 0.7 mM EDTA in Dulbecco’s phosphate buffered saline (pH 7.4), and then for 2–5 minutes in 0.05% trypsin. Cells were then washed with phosphate buffered saline/EDTA and used for experiments, or passaged in a ratio appropriate to the culture’s growth rate. Cells were cultured from passages 3 to 7 (depending on the passage number of the original stock) to passage 14. No passage-dependent metabolic changes were observed in any of the cell lines. All experiments were performed in serum free media.

Alternatively, 25 ml of peripheral blood was collected into heparinized tubes by venous puncture and PBMCs were purified by histopaque 1077. PBMCs were archived in 90% fetal calf serum/10% DMSO at -80 or liquid nitrogen and analyzed within 3 months, then thawed and cultured as described [25].

### Stimulation conditions

TNFα (Genzyme Corporation, Cambridge, MA) was used at concentrations that approximate physiological conditions (10 ng/ml: 60 pM, or 25 ng/ml: 150 pM) as indicated. Cells were pre-incubated with TNFα in serum-free medium for 24 hr prior to the indicated analyses. Cells treated with FFAs were additionally/alternatively incubated for 24 hrs in oleic acid complexed to bovine serum albumin (BSA) at a molar ratio of 3:1 and a concentration of 2 mM. For assays that cannot be completed in the presence of BSA for technical reasons, cells were treated with oleate (25 or 50 μM) complexed to the alternate delivery molecule cyclodextrin [26] in a 1:6 molar ratio.

### Lipid accumulation

Fibroblasts were plated in 48-well plates at the indicated densities then stimulated with 2 mM oleate prior to staining with Nile Red (1 μg/ml). PBMCs were plated on poly-D-lysine (PDL) coated 6-well plates at a concentration of 10^6^ cells per well and stained with Nile Red. Cells were photographed using a Nikon Eclipsed TE200 (Melville, New York) and analyzed on a Tecan Microplate Reader (Männedorf, Switzerland) at excitation/emission wavelengths of 489/560 nm. Data were normalized on a per cell basis and expressed as a percentage of the values for unstimulated cells.

### Long-chain acyl-CoA (LC-CoA)

Fibroblasts were plated at 7 X 10^5^ cells per well in a 12-well plate and incubated with or without oleate. The following day, cells were washed with modified Krebs buffer, incubated in a 1% trichloroacetic acid solution containing 3.75 mM DL-dithiothreitol (DTT) for 10 minutes, then precipitates were washed with water. Precipitates were hydrolyzed for 10 minutes in 300 μL of 3.75 mM potassium phosphate buffer (pH = 11.2) to convert LC-CoA to free CoA and fatty acid. The hydrolysis buffer was neutralized with 2.1 mM TRA-HCl to a pH of 7.4, and free CoASH was measured enzymatically by utilizing the α-ketoglutarate dehydrogenase reaction [27]. Standard curves were produced by serial dilution of hydrolyzed long-chain acyl-CoA (palmitoyl- CoA). NADH luminescence was measured on a Tecan Microplate Reader at an integration time of 200 milliseconds as described previously [28].

### Fatty acid oxidation (FA-oxidation)

Fibroblasts were plated in 24-well plates at a density of 2 X 10^5^ cells per well and exposed to TNFα for 24 hours. Following TNFα exposure, ^14^C-oleate oxidation assays for measuring fatty acid conversion to ^14^CO_2_ were performed as previously described [29]. Briefly, cells were incubated in 500 μl/well of modified Krebs buffer containing 3 mM glucose and 12.5 μM ^14^C-oleate (54 mCi/mmole, Perkin Elmer). A 1.5 cm round filter paper (Whatman) was suspended above each well and the plate was sealed for a 2 hr incubation period. At the end of the incubation period, β-phenylethyl amine was injected onto the filter paper, followed by acidification of the media with 100 μl/well of 6M sulfuric acid. The cell plate remained sealed for an additional hour to trap evolved ^14^CO_2_ onto the filters. Filter papers were counted in scintillation fluid (Ecoscint, National Diagnostics) and β particle emission was analyzed using a LabLogic 300SL Liquid Scintillation Counter (Brandon, Florida). For some studies, FA oxidation was blocked with etomoxir (30 αm), which inhibits FA activation to LC-CoA by CPT1 and thus, prevents FA entry into the mitochondria and subsequent oxidation.

### NAD(P)H autofluorescence

NAD(P)H fluorescence was determined by exciting at 340 nm and measuring emission at 460 nm using an Hitachi F-2000 fluorescence spectrophotometer (Hitachi High Technologies Corp., Tokyo, Japan). Following trypsinization and washing with PBS/EDTA, approximately 3.5 X 10^5^ cells were suspended in modified Krebs-HEPES buffer containing 2 mM glucose at pH 7.4. Maximum oxidization of cells was induced by addition of 2.5 μM FCCP, and full reduction by addition of 10 mM cyanide. Values were expressed as a percentage of maximum reduction.

### O_2_ consumption

O_2_ consumption was measured using the Seahorse XF24 flux ion analyzer (Boston, Massachusetts). Fibroblasts were plated on 24-well Seahorse plates at a density of 1.5 X 10^5^ cells per well. TNFα was added to some wells 3 hrs after attachment. The plate was then incubated at 37°C with 5% CO_2_ overnight. Media were then aspirated from the plate and cells were incubated in Krebs buffer containing 15 mM glucose and 10 mM each of glutamine and leucine for 1 hour. During the measurement phase, four solutions were automatically injected into each well at indicated times. The first injection (A) was a Krebs solution containing 10 mM glutamine and 10 mM leucine plus oleate (0–50 μM) followed by sequential injections of 10 μM oligomycin-A (B), 2.5 μM FCCP (C) and 10 μM antimycin-A (D). To account for variation in initial OCR values among samples, the ratio of each test condition’s OCR was normalized to the control OCR by calculating a correction factor used to adjust each data point.

### Lipid peroxidation (LPO)

LPO was assessed in fibroblasts based on an established protocol [30]. LPO was alternatively assessed in the PBMCs used in the lipid accumulation experiments (1 million cells per PDL-coated well). Briefly, cells were plated at a density of 3 X 10^4^ cells per well and grown to confluence in a 48-well plate, then stimulated with oleate and/or TNFα before exposure to a 0.4% 2-thiobarbituric acid/10% acetic acid solution. Sodium hydroxide was added to achieve a final concentration of 0.0625 N. Standard curves were produced by serial diluting 1,1,3,3-tetraethoxypropane (Sigma, St. Louis, MO). The cell solution was incubated at 90°C for 60 min, then cooled and centrifuged at 15,000 g for five minutes. The supernatant was isolated and fluorescence was measured on the Tecan M1000 plate reader (Männedorf, Switzerland) with an excitation wavelength of 515 nm and an emission wavelength of 553 nm. Due to variation [31] among experiments, data were analyzed as a percentage of unstimulated control.

### Statistics

Data are presented as the mean and SEM of 6 experiments per condition unless otherwise specified. Two-way ANOVA with Tukey post-hoc analysis was used to determine significant differences (*p*<0.05) among groups [31].

In all figures, * indicates *p* < 0.05, ** indicates *p* < 0.01, *** indicates *p* <0.001.

## Results

### T1D cells accumulate more lipid than control cells

**Fig 1:**
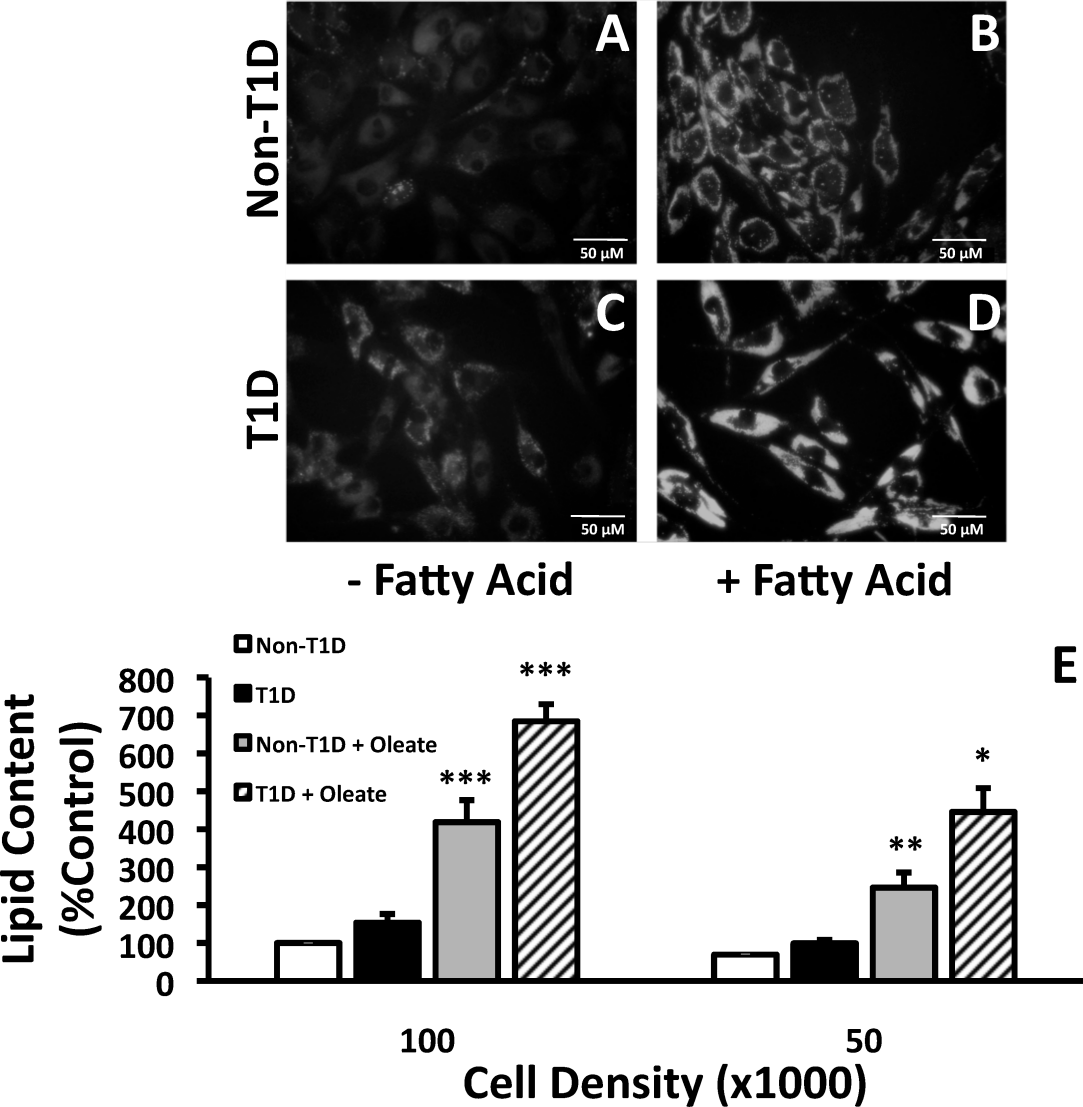
Lipid accumulation in human fibroblasts incubated with oleate. Representative images of fibroblasts after staining with Nile Red are shown in Panels A-D. A and B represent non-T1D fibroblasts with (B) or without (A) stimulation with oleate. Panels C and D represent T1D fibroblasts with (D) and without (C) FA stimulation. (E) Lipid quantitation by image analysis of the Nile red stain. Human skin fibroblasts from 3 separate non-T1D and T1D patients were plated at indicated cell densities and stained with Nile Red after incubation with 2 mM oleate complexed to BSA in a 3:1 ratio for 24 hours. Data (E) are presented as a percent of control (Non-T1D, untreated cells) calculated from a Tecan Microplate Reader.

**Fig 2:**
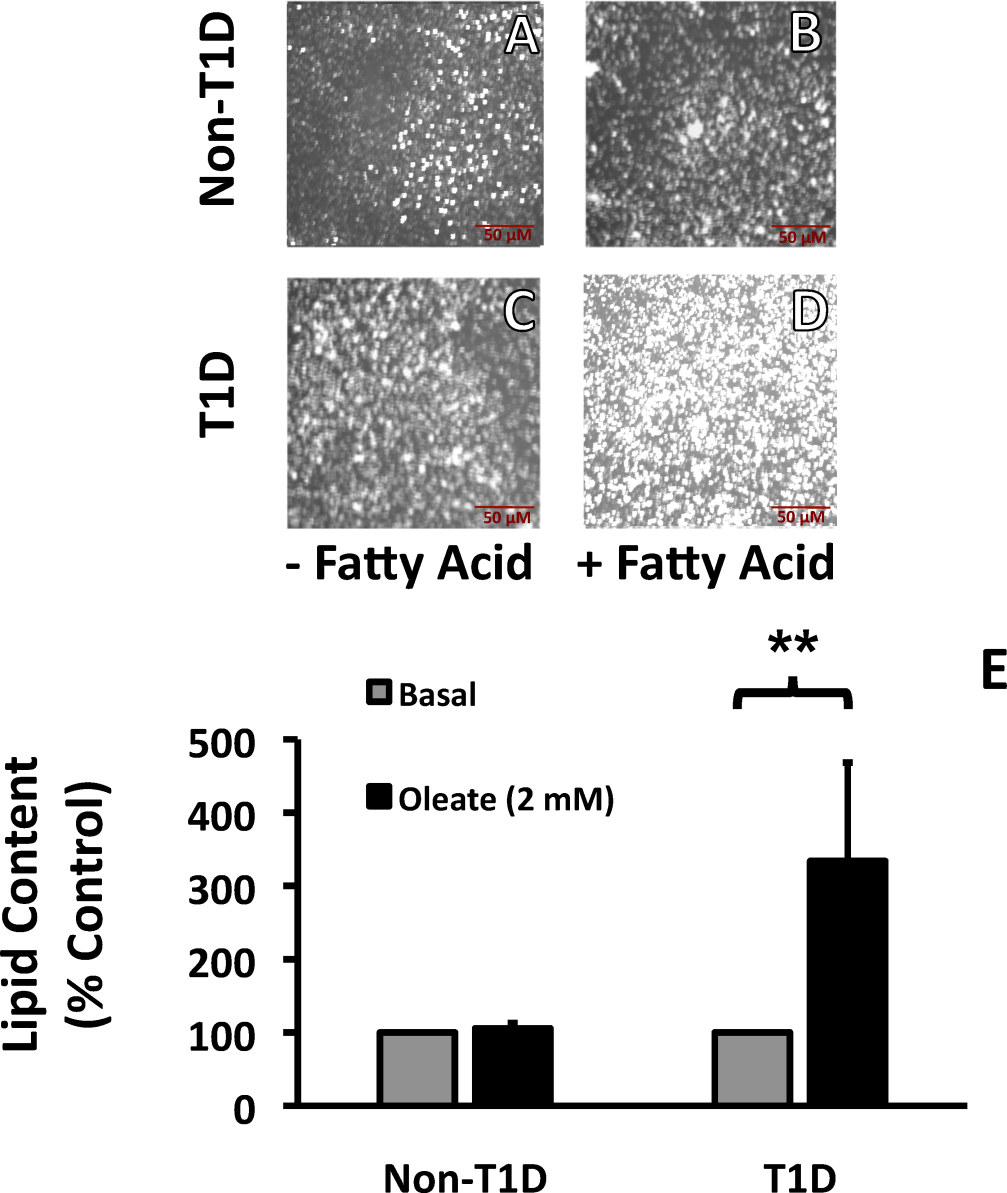
Lipid accumulation in human PBMCs incubated with oleate. PBMCs were plated on PDL-coated plates ± oleate (2mM) complexed to BSA in a 3:1 ratio for 24 hr before being stained with Nile Red. Data are presented as a percent of control (Non-T1D, untreated cells) calculated from a Tecan Microplate Reader. N = 4.

Lipid content is an indicator of FA activation and subsequent storage, rather than oxidation. Although both T1D and control fibroblasts increase lipid content following FA incubation (Fig 1, compare A to B and C to D), fibroblasts from T1D subjects accumulated more lipid than fibroblasts from control subjects under both control (Fig 1C vs 1A) and FA-stimulated conditions (Fig 1D vs 1B), and lipid accumulation was largely independent of plating density (Fig 1E). In contrast, TNFα did not alter lipid content in either control or T1D fibroblasts (data not shown). Results from lipid accumulation studies in PBMCs were somewhat different: lipid levels were similar in PBMCs from control and T1D subjects in the absence of stimulation Fig 2, left panels), and only PBMCs from T1D subjects increased lipid content in response to FA (Fig 2, right panels). Taken together, these data indicate that lipid storage is higher in both fibroblasts and immune cells from T1D subjects.

### T1D fibroblasts contain more LC-CoA than controls after chronic exposure to oleate

**Fig 3:**
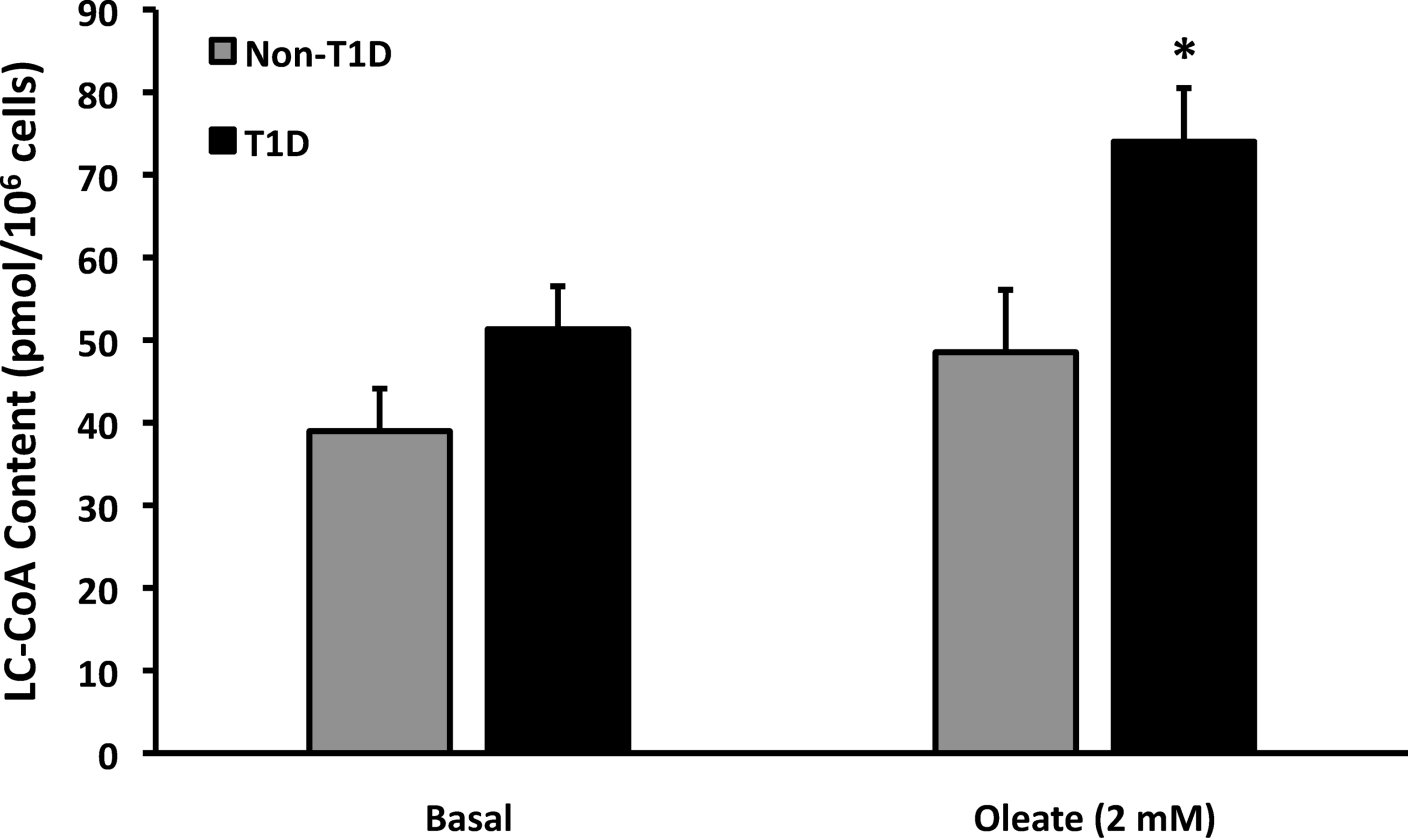
LC-CoA content in human fibroblasts. LC-CoA content was determined following culture of fibroblasts ± 2 mM oleate complexed to BSA in a 3:1 ratio for 18 hours. T1D fibroblasts, on average contained 74 ± 6.5 pmoles/million cells whereas non-T1D fibroblasts contained 49 ± 7.6 pmoles/million cells, following oleate exposure (N = 6).

The first step in cellular metabolism of FA is activation to LC-CoA via long-chain acyl CoA synthetases. We therefore measured LC-CoA to determine whether the increased T1D lipid content was associated with increased FA activation in T1D fibroblasts. Fig 3 shows that no significant difference was observed without added oleate. Oleate exposure led to an insignificant increase in LC-CoA in non-T1D fibroblasts, but a significant 53% increase in LC-CoA in fibroblasts from T1D subjects. These data suggest that the greater lipid content in FA-exposed fibroblasts from T1D subjects was either due to increased lipid synthesis via LC-CoA or decreased ß-oxidation.

### Fibroblasts from T1D subjects converted more FA to CO_2_ than control fibroblasts

**Fig 4:**
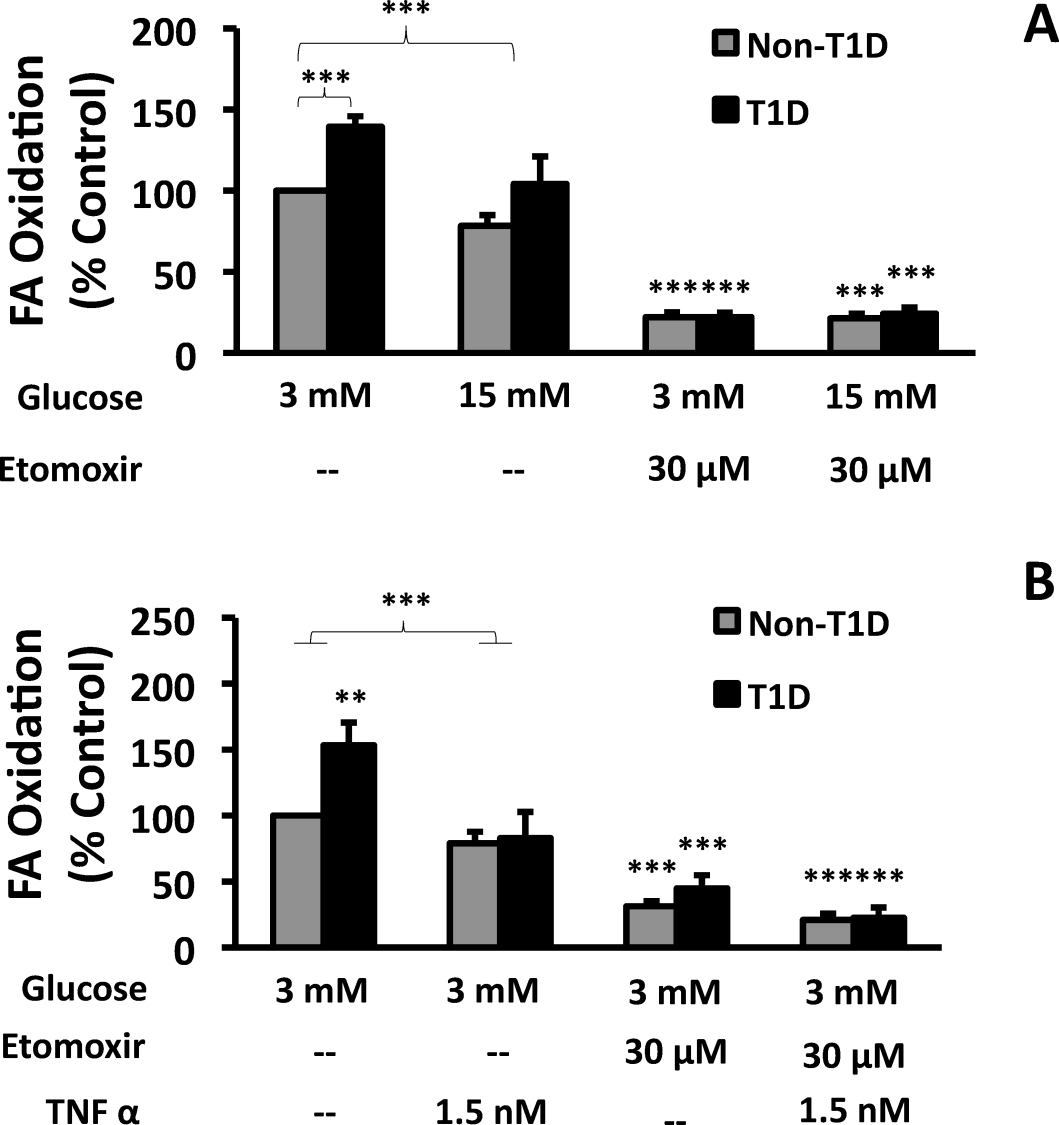
The effect of glucose and TNFα on FA oxidation in human fibroblasts. Non-T1D and T1D fibroblasts were exposed to ^14^C-labeled oleate for 2 hours in the presence of either 3 mM or 15 mM glucose (A) or after 24 hr exposure to 1.5 nM TNFα (B) and the conversion of labeled oleate to ^14^CO_2_ during the 2 hr incubation was measured as described in Methods. Etomoxir, an inhibitor of CPT1 and FA oxidation, was used as a negative control. Data are presented as a percentage of control fibroblast FA oxidation (Non-T1D, untreated cells) (N = 6).

To determine whether higher LC-CoA levels in FA-exposed cells from T1D subjects (Fig 3) were due to low FA-oxidizing capacity, causing increased partitioning to stores, we measured flux of ^14^C-labeled FA to ^14^CO_2_. We further compared control conditions (3 mM glucose) and high glucose (15 mM) to assess mitochondrial flexibility to use either glucose or FA as substrate. At 3 mM glucose, FA oxidation was higher in T1D cells than non-T1D cells (Fig 4A, left bars). Acute exposure (2.5 hours) to high glucose (15 mM) inhibited FA conversion to CO_2_ similarly (21–22%) in both control and T1D fibroblasts (Fig 4A). The positive control, as expected, was markedly inhibited by etomoxir (Fig 4A, right bars). Taken together, these data showed that T1D fibroblasts oxidized more FA than non-T1D cells, indicating that T1D cells were not defective in FA oxidation, but rather have both increased lipid synthetic and oxidative capacity.

### TNFα inhibits FA conversion to CO_2_ in fibroblasts

TNFα has been shown to inhibit FA oxidation in other cell types [32]. To determine whether TNFα impacted FA oxidation in cells from T1D and non-T1D subjects, we incubated cells with 150 pM TNFα and 3 mM glucose for 24 hr and measured FA oxidation. TNFα treatment significantly diminished FA oxidation in fibroblasts from T1D subjects, but not from non-T1D subjects (Fig 4B, left bars). As expected, etomoxir blocked FA oxidation under all conditions tested (Fig 4A and 4B, right bars). We conclude that TNFα eliminated the elevated FA oxidation characteristic of fibroblasts from T1D subjects.

### TNFα induced a more oxidized state in T1D fibroblasts

**Fig 5:**
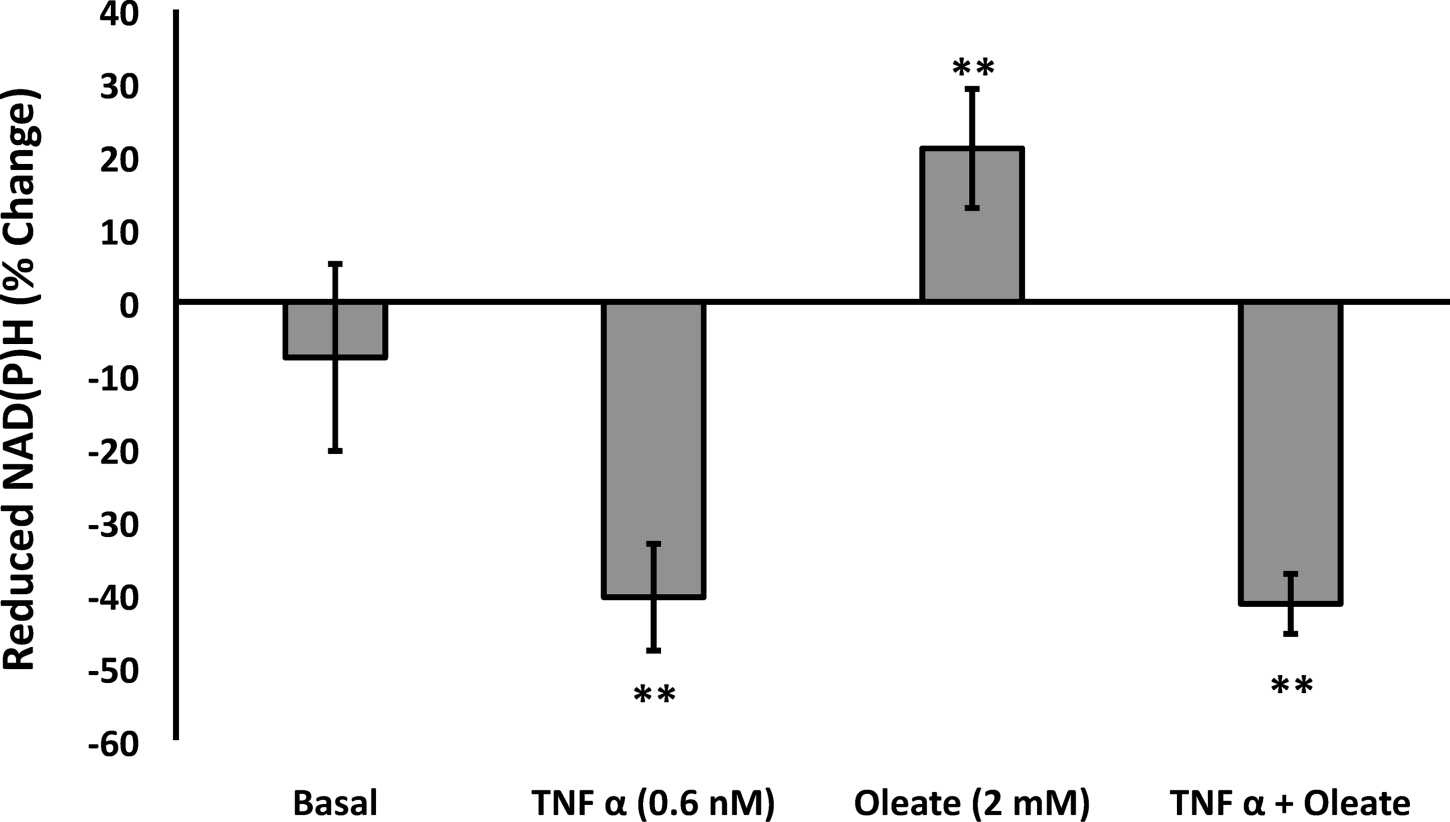
Fibroblasts from T1D subjects reacted to FA and TNFα with altered cellular redox state. The redox state of fibroblasts was measured using NAD(P)H autofluorescence after a 24 hr exposure to 2 mM oleate, 0.6 nM TNFα, or both. Data are presented as a percent change from the control state on a scale ranging from fully oxidized (caused by 2.5 μM FCCP and considered 0% reduced) to fully reduced (caused by 10 mM cyanide and considered 100% reduced). In non-T1D subjects control percent reduced averaged 38% of maximum. No significant differences were observed under control conditions between the T1D and non-T1D cells (left bar). The right 3 bars indicate that significant change occurred in response to FA and TNFα in T1D subjects. No significant changes were occurred in non-T1D fibroblasts in response to TNFα and/or oleate (not shown) (N = 6).

FA conversion to CO_2_ causes a more reduced mitochondrial redox state due to production of NADH during FA β-oxidation [33]. To determine whether the changes in oxidation of FA to CO_2_ in the presence and absence of TNFα altered the redox state in human fibroblasts, we quantified the percentage change of pyridine nucleotide reduction under different incubation conditions, based on a scale of 100%, from fully reduced with cyanide, to 0%, fully oxidized with the uncoupler, FCCP. Cyanide-mediated reduction was set to 100% reduced, and the fully oxidized state was determined by exposing the cells to FCCP (0% reduced, 100% oxidized). Fibroblasts from non-T1D subjects were 37% reduced and showed small and insignificant changes in redox state in response to oleate and TNFα (data not shown). There was also no signifcant difference between non-T1D and T1D cells under control conditions (Fig 5, left bar). In contrast, in T1D fibroblasts, incubation with TNFα oxidized the mitochondrial redox state by an average of about 40% (Fig 5, second bar) while oleate reduced the redox state by about 20% (Fig 5, third bar). The redox state in T1D cells treated with a combination of oleate and TNFα was dominated by the TNFα effect and was more oxidized by about 40%, consistent with the observed ability of TNFα to inhibit FA oxidation [32] and thus prevent the production of mitochondrial reducing equivalents during β-oxidation. It should be noted here that Fig 5 reflects changes in steady state not flux and provides no information about possible opposing reactions such as utilization of electrons from NADH for oxidative phosphorylation, proton leak or transhydrogenation.

### T1D cells preferentially consume O_2_ to make ATP with less proton leak than non T1D cells

**Fig 6:**
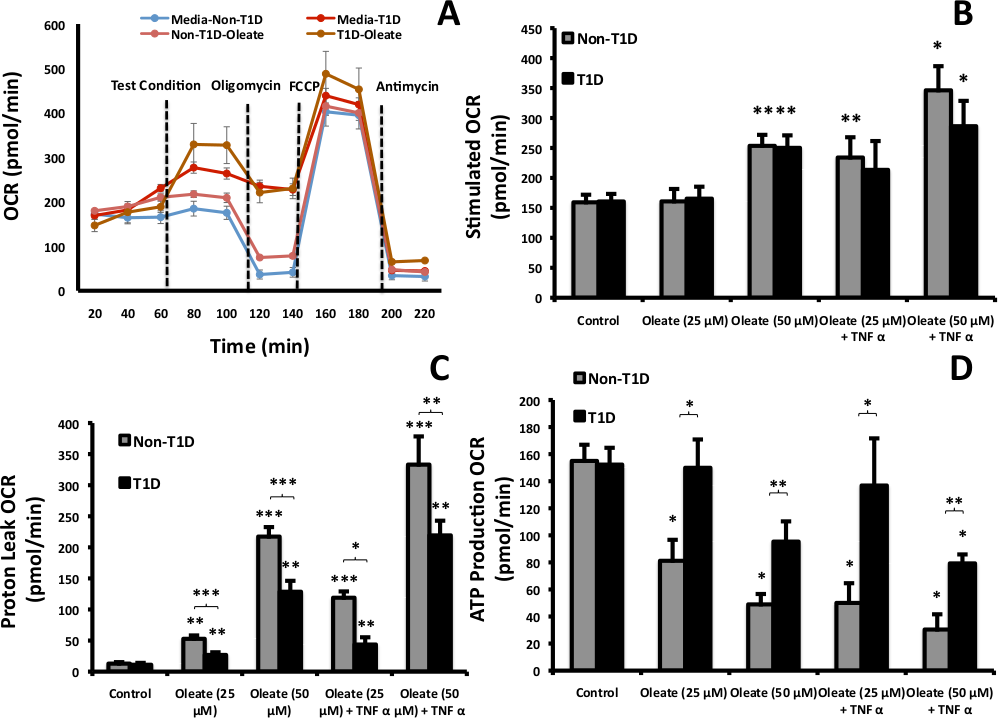
The effect of FA and TNFα on O_2_ consumption rate (OCR) in human fibroblasts. (A) Illustration of OCR time course traces of T1D and non-T1D fibroblasts after acute exposure to 50 μM oleate. (B) Total OCR ± either 25 or 50 μM oleate complexed to cyclodextrin and ± 24 hr exposure to TNFα. (C) The proton leak was calculated by subtracting the difference between OCR after exposure to antimycin A (totally inhibited respiration) and oligomycin (ATP generating respiration). (D) OCR used to produce ATP was calculated from the decrease induced by oligomycin addition (N = 6).

To directly compare mitochondrial fluxes in respiration in T1D and control fibroblasts, we quantified the effects of oleate and/or TNFα exposure on O_2_ consumption rate (OCR) by cells from T1D or non-T1D subjects using extracellular flux analysis. To determine efficiencies in respiration, mitochondrial OCR can be separated into two components: (1) O_2_ used for ATP production (oligomycin-sensitive) and (2) mitochondrial proton leak (the difference between Antimycin A-(fully inhibited) and oligomycin-inhibited respiration) as illustrated in Fig 6A. Fibroblasts were acutely exposed (18 mins) to oleate complexed to the vehicle (cyclodextrin) or following 24 hr pre-treatment with TNFα. OCR by fibroblasts did not change in response to 25 μM oleate, but increased in response to 50 μM oleate (Fig 6B). TNFα increased OCR compared to oleate alone in both T1D and non-T1D cells (Fig 6B). Proton leak was induced by oleate in cells from both cohorts, but was significantly lower in fibroblasts from T1D subjects under all conditions tested (Fig 6C). Lower proton leak in T1D fibroblasts corresponded with higher ATP production relative to non-T1D cells, in part because ATP production by fibroblasts from non-T1D subjects was significantly decreased by oleate and/or TNFα exposure (Fig 6D). Since total OCR was similar and FA-conversion to CO_2_ was higher in T1D compared to control fibroblasts (Fig 4A and 4B), mitochondrial measurements are consistent with the interpretation that T1D fibroblasts were more efficient in oxidizing lipid with less proton leak, and thus were able to provide the additional ATP needed to activate and store more FA (Fig 1) without requiring additional oxygen.

### T1D cells contained more peroxidized lipid than controls

**Fig 7:**
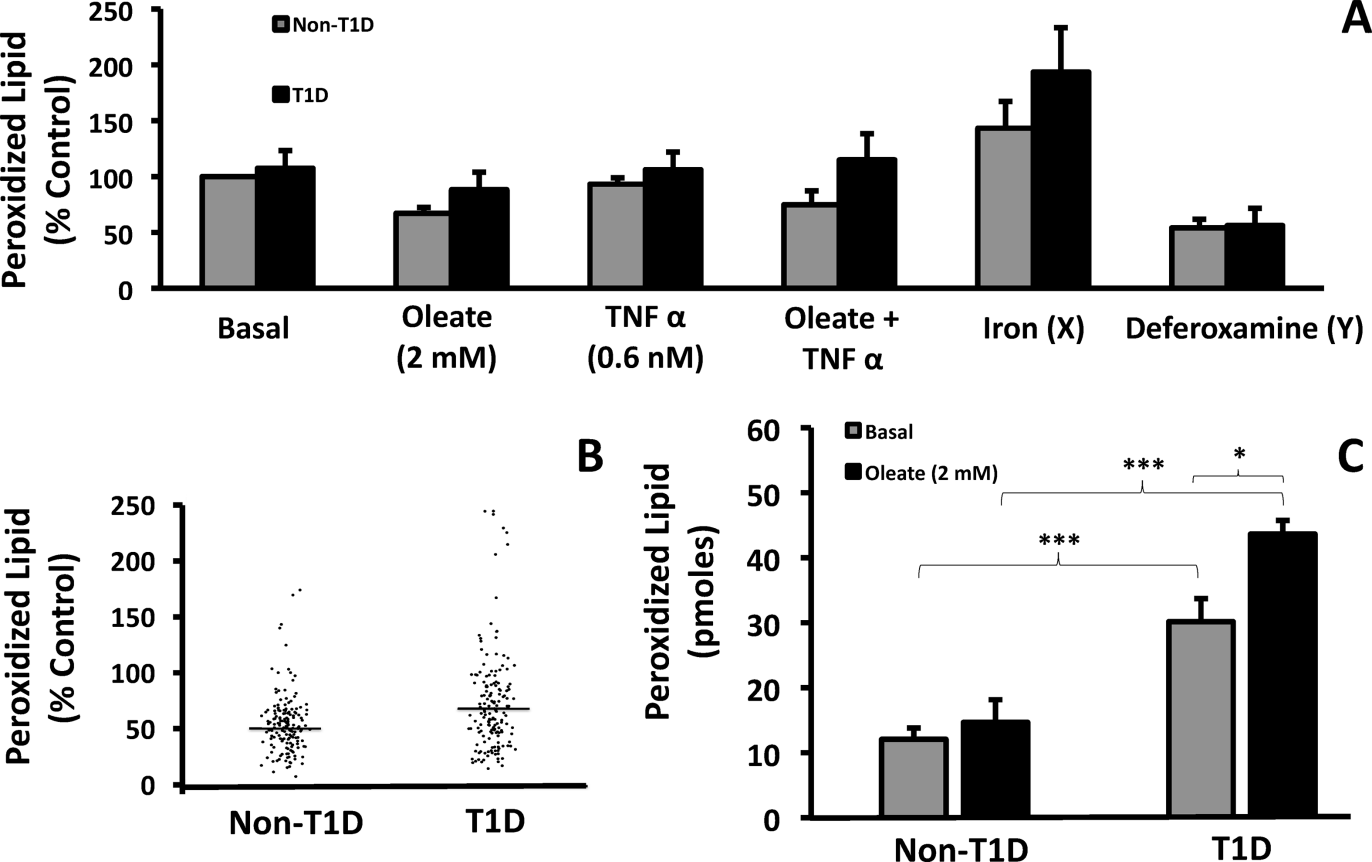
Lipid peroxidation in human fibroblasts and PBMCs. The amount of peroxidized lipid (LPO) was measured using the thiobarbituric acid reaction assay. (A) Comparison of LPO in non-T1D and T1D fibroblasts (N = 7). (B) Scatter graph of all control and T1D values plotted along with the mean of the pooled data points (horizontal lines) (N = 7). While there was no significance between control and T1D peroxidized lipid under the individual test conditions in panel A, ANOVA analysis revealed that when all results from panel A were pooled, T1D fibroblasts have a significantly higher amount of peroxidized lipid than controls. (C) LPO content in PBMCs from non-T1D and T1D patients (N = 4).

Diminished proton leak with higher flux implicates increased production of reactive oxygen species (ROS) via the electron transport chain. To test this possibility, we assessed peroxidized lipid, a consequence of ROS production in cells in the presence and/or absence of oleate and/or TNFα. Control experiments validated that iron (100 μM) increased and deferoxamine (50 μM) decreased peroxidized lipid, respectively, as expected (Fig 7A, right panels). However, peroxidized lipid was not significantly increased by oleate and/or TNFα in any of the fibroblasts, nevertheless, ANOVA analysis of all pooled conditions showed that fibroblasts from T1D had more peroxidized lipid than fibroblasts from non-T1D subjects (Fig 7B, 105 ± 4.5 pmol vs. 82 ± 4.5 pmol, *p* < 0.0005). In contrast, PBMCs from T1D subjects had increased amounts of peroxidized lipid, regardless of FA treatment, compared to PBMCs from non-T1D subjects (Fig 7C). Oleate treatment further increased peroxidized lipid only in PBMCs from T1D subjects.

## Discussion

Our data provide strong evidence both with fibroblasts and PBMCs to support the conclusion that cells from T1D subjects handle FA differently from non-T1D cells as regards fat storage, LC-CoA content, oxidation, and energy efficiency of ATP production. Some differences were only revealed in the presence of added FAs (documented using oleate) or an inflammatory mediator (as modeled by TNFα). Additional work is needed to identify the molecular basis for these differences. Specifically, additional PBMC and fibroblast samples need to be compared within a closer age range. The average age of the T1D fibroblast donor was 19 years old while the average age of the PBMC sample donor was 33 years old (S1 Table). This age difference could be a confounding variable that influenced the overall metabolism of the cells. Additionally, it is important to differentiate amongst three putative mechanisms to explain the increased LC-CoA and lipid accumulation in T1D cells: (1) cellular trapping of FA in cytosolic LC-CoA due to increased expression, activity or localization of acyl-CoA synthetases (ACS), (2) increased cytosolic acyl CoA or fatty acid binding proteins (FABP) or (3) preferential triglyceride synthesis relative to FA oxidation. Recent evidence has shown that there is a strong correlation between T1D incidence and up-regulation of FABP-5 [3] that favors triglyceride synthesis [34].

These findings, coupled with our previous work showing differences in Ca^2+^ mobilization in T1D compared to non-T1D fibroblasts [35], support the likelihood of metabolic differences in both non-immune and immune cells in T1D, which may both impact and be impacted by the virally-induced inflammation that frequently precedes overt T1D [36, 37].

Our data support increased mitochondrial efficiency in T1D that could be either beneficial or harmful. In many cells, proton leak is hypothesized to be a beneficial adaptive response that uses the proton gradient to generate the NADPH needed to scavenge ROS and minimize oxidative damage [38–40]. Electrons used to convert NADH to NADPH appear as a leak since the nicotinamide nucleotide transhydrogenase (NNT), like ATP synthase, uses the mitochondrial proton gradient as an energy source [41]. The lower proton leak in T1D cells with FA as a fuel source is associated with greater mitochondrial efficiency since the proton gradient is used for ATP synthesis rather than for ROS scavenging. Such a reduction in leak could then lead to an increase in ROS production in T1D cells. Because ROS production requires a high mitochondrial membrane potential that is decreased by a proton leak [42–44], the lower proton leak in T1D cells may explain the higher amounts of peroxidized lipid in PBMCs and fibroblasts from T1D compared to non-T1D subjects under various conditions.

Based on our results, we propose a model wherein differences in metabolism in cells from T1D subjects react to exposure to FA by increasing LC-CoA and triglycerides and generate a more reduced state of the pyridine nucleotides within the cell (Figs 2 and 3). The prevention of excess ROS through proton leakage in non-T1D cells exposed to FA but not in T1D cells could underlie differences in ROS and lipid peroxidation (Fig 7).

Overall, our demonstration of metabolic differences in T1D cells sets the stage for the identification of molecular mechanisms underlying these differences in cellular function and highlights a promising new avenue of inquiry into metabolic contributors to T1D that could apply broadly and inform preventive strategies.

## Supporting information

S1 Table T1D donor treatment information. Information regarding the age, sex and treatment at time of donation of all T1D donors (fibroblasts and PBMCs).
(TIF)
