# Supplementary figures and images for: Type 1 diabetes alters lipid handling and metabolism in human fibroblasts and peripheral blood mononuclear cells
Source: PLoS One. 2017 Dec 4;12(12):e0188474. doi: 10.1371/journal.pone.0188474 (PMC5714353; doi:10.1371/journal.pone.0188474)

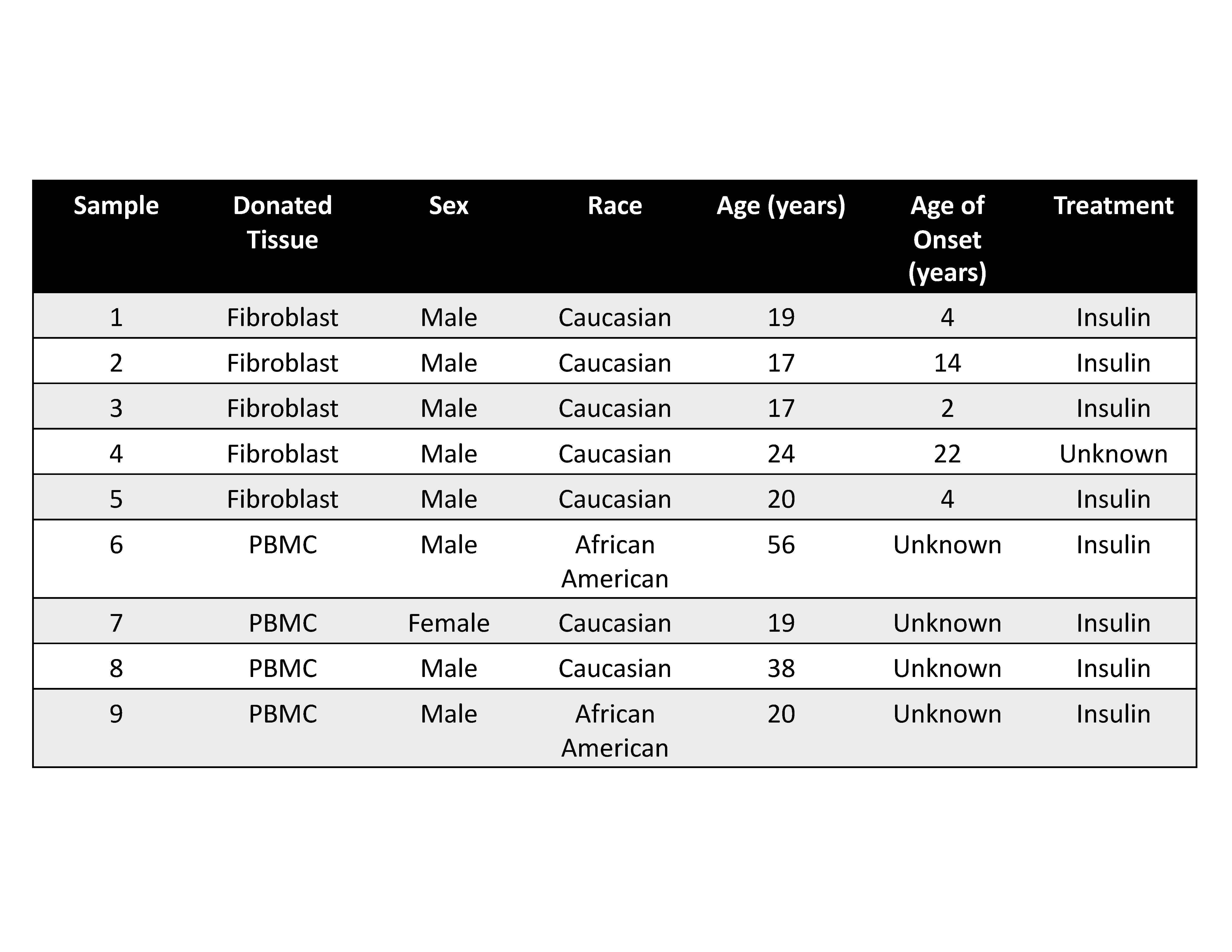

Supplement: S1 Table — Information regarding the age, sex and treatment at time of donation of all T1D donors (fibroblasts and PBMCs). (TIF) [file pone.0188474.s001.tif]
